# 3D Galileo Reference Antenna Pattern for Space Service Volume Applications

**DOI:** 10.3390/s24072220

**Published:** 2024-03-30

**Authors:** Francesco Menzione, Matteo Paonni

**Affiliations:** European Commission, Joint Research Centre (JRC), 21027 Ispra, Italy; matteo.paonni@ec.europa.eu

**Keywords:** GNSS, space service volume, Galileo antenna pattern, antenna characterization, spherical harmonic, statistical learning, elastic-net regularization, high-orbit navigation, accessibility index

## Abstract

There is an increasing demand for navigation capability for space vehicles. The exploitation of the so-called Space Service Volume (SSV), and hence the extension of the Global Navigation Satellite System (GNSS) from terrestrial to space users, is currently considered a fundamental step. Knowledge of the constellation antenna pattern, including the side lobe signals, is the main input for assessing the expected GNSS signal availability and navigation performance, especially for high orbits. The best way to define and share this information with the final GNSS user is still an open question. This paper proposes a novel methodology for the definition of a high-fidelity and easy-to-use statistical model to represent GNSS constellation antenna patterns. The reconstruction procedure, based on antenna characterization techniques and statistical learning, is presented here through its successful implementation for the “Galileo Reference Antenna Pattern (GRAP)” model, which has been proposed as the reference model for the Galileo programme. The GRAP represents the expected Equivalent Isotropic Radiated Power (EIRP) variation for the Galileo FOC satellites, and it is obtained by processing the measurements retrieved during the characterization campaign performed on the Galileo FOC antennas. The mathematical background of the model is analyzed in depth in order to better assess the GRAP with respect to different objectives such as improved resolution, smoothness and proper representation of the antenna pattern statistical distribution. The analysis confirms the enhanced GRAP properties and envisages the possibility of extending the approach to other GNSSs. The discussion is complemented by a preliminary use case characterization of the Galileo performance in SSV. The accessibility, a novel indicator, is defined in order to represent in a quick and compact manner, the expected Galileo SSV quality for different altitudes and target mission requirements. The SSV characterization is performed to demonstrate how simply and effectively the GRAP model can be inserted into user analysis. The work creates the basis for an improved capability for assessing Galileo-based navigation in SSV according to the current knowledge of the antenna pattern.

## 1. Introduction

Use of global navigation satellite systems (GNSSs) in the Space Service Volume (SSV), and particularly for certain missions (MEO, GEO, HEO, Lunar, and beyond), requires knowledge about the GNSS transmission antenna patterns, or a representative model thereof, including side lobes [1]. Different studies of navigation in high orbit ([2,3]) rely on the knowledge of the received signal power, which is instrumental for a wide range of link budget analysis used for spaceborne receiver design, navigation performance assessment and implementation of filtering techniques of the GNSS observables [4,5,6,7]. The best way to retrieve and share this information with the final GNSS user is still an open research issue. The availability of the antenna pattern measurements at system level can be sparse and prone to limitation of the testing facilities. In fact, different pattern realizations can be experienced, even with the same antenna design. This generally corresponds to a burden for users, who still need to aggregate the data ([2,3]) in order to mitigate the discontinuities and measurement degradation effects. This paper proposes a novel approach aiming to achieve the same objectives by considering a more general representation, which preserves all the features of the constellation antenna pattern with improved statistical significance. This derives from a data-driven model reconstruction, providing antenna pattern expected mean and variance distributions. Such an approach is here implemented and adopted for the definition of the Galileo Reference Antenna Pattern (GRAP), for which metadata have recently been published in [8]. The proposed reconstructed model is an efficient and compact tool to provide the GNSS user and community with the most stable representation of the expected Galileo FOC transmitting antenna pattern, according to the knowledge matured at system level before the deployment of the service. The paper aims at providing a more in-depth presentation of the methodologies put in place for the model development, and showing how it can be easily exploited to perform a preliminary characterization of the expected Galileo SSV. 

The first contribution is a detailed description of the GRAP model synthesis [8]. The procedure ingests the information retrieved during the antenna characterization campaign performed on the Galileo FOC satellite up to the highest available off-boresight angles, before their integration on the satellites. Instead of just pooling all observations to achieve a better statistical representation of the expected system performance, an ad-hoc multi-step procedure has been developed. The approach aims at optimally aggregating the available antenna pattern information using methodologies derived from antenna characterization techniques [9,10,11,12,13,14] and statistical learning [15]. The first step consists of a frequency resample of the power distribution. After that, a reconstruction algorithm based on representation of the pattern in a sparse spherical harmonic basis is considered. The algorithm exploits an elastic net regularization [16,17] in order to improve the smoothness of the 3D spatial distribution and obtain its representation in sparse spherical harmonic parameter space. The final step introduces the concept of representing the constellation antenna pattern as an hierarchical population model [18]. The reconstructed patterns from different data collections are addressed as different realizations of the same model and merged, in order to achieve a general and easy-to-use representation of the antenna for the final GNSS space user. One of the most relevant features of the proposed statistical process is the possibility to define the GRAP values and their correspondent confidence bounds, so the idea is to complement the antenna pattern metadata [8] with information representative of the uncertainty level associated with the different pattern realizations. This level of confidence can be reflected in the user’s analysis; hence, their conclusions inherit the capability to assess what will likely happen according to current knowledge of the pattern. For the sake of brevity and without loss of generality, all the results presented hereafter refer to the Galileo E1 carrier frequency, but extension to other Galileo frequencies can be easily derived by using the metadata provided in [8] and adjusting all frequency-dependent parameters.

The second contribution of this paper is to process the developed GRAP model into a preliminary use case characterization of the Galileo SSV performance. Such an assessment is based on a simplified SSV geometrical model allowing easy definition of a target user altitude, the GRAP region spanned, and the contribution to the overall received signal power. More importantly, the proposed SSV analysis introduces a novel compact Key Performance Indicator (KPI), namely the accessibility. The introduced KPI expresses the effective arc of a rising GNSS Space Vehicle (SV) that can be exploited for navigation purposes, considering a target received power or receiver sensitivity. The results show that this parameter, even though limited by the selected geometry and signal propagation assumptions, provides a new, competitive and compact performance index to assess antenna pattern-driven GNSS SSV peculiarities. 

The paper is organized as follows. Section 2 defines the general constellation antenna pattern reconstruction procedure and its mathematical framework. The resulting 3D GRAP is presented in Section 3 and complemented with the analysis of properties deriving from the reconstruction procedure. In Section 4.1, the simplified SSV model and accessibility index are introduced. The information about the GRAP is injected into the SSV model and, in Section 4.2, a Galileo SSV characterization is provided in terms of accessibility charts. Section 5 assesses the general conclusions and identifies the way forward for the current and future improvement of the Galileo SSV.

## 2. Multi-Step 3D Reference Antenna Pattern Reconstruction

The GRAP model is the result of processing of the dataset made available by the Galileo programme [8] and retrieved from several testing campaigns performed on the Galileo FOC satellite’s isolated antennas, i.e., before their integration on the satellite.

**Figure 1:**
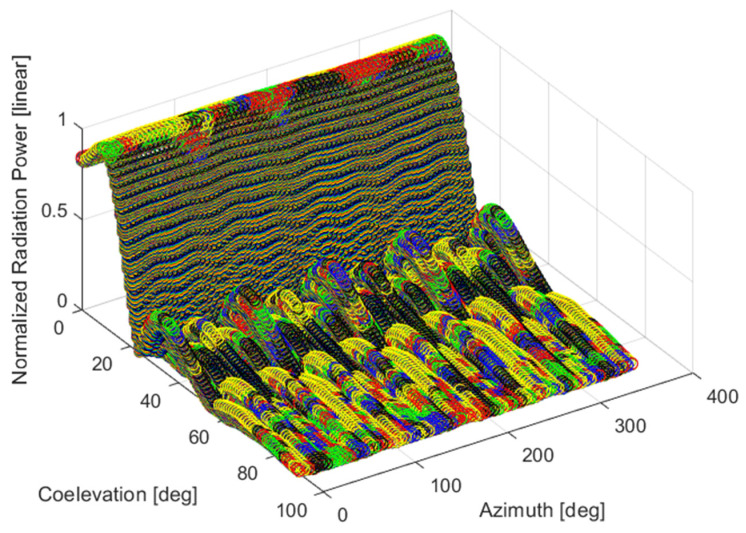
Rapresentation of Continuous Wave (CW) spherical scanning of the antenna pattern radiated field: different colors represent data samples collected at different central frequencies spaced 2.5 MHz apart.

The measurement dataset has been provided as Continuous Wave (CW) spherical scanning of the radiated field for a target antenna i. The pattern measurements are indicated as

(1) $$G^{i}\left(\left.\theta ,\varphi ,f_{CW}\right|\theta_{1},\varphi_{1},f_{1},\theta_{2},\varphi_{2},f_{2},\ldots \theta_{n},\varphi_{n},f_{n}\right)$$

where $G^{i}\in \left[0,\;1\right]$ represents the radiated field normalized power in linear scale for the *i*-th antenna (Figure 1), measured at the different co-elevation $\theta_{j}\in [35,90{}^{\circ}]$ and azimuth $\varphi_{k}\in [0,360{}^{\circ}]\;$ scanning points for a fixed CW frequency $f_{CW}$.

The reference antenna pattern derivation is here intended as a data processing approach aiming to achieve the following three objectives:

(1) Provide to the user a representation of the pattern applicable to target Galileo signals, hence the need to report the measurement at different CW to the Galileo central frequencies.
(2) Overcome the facility sampling limitations and achieve a high-resolution 3D antenna pattern with smooth distribution, filtering out discontinuities and measurement degradation.
(3) Handle the uncertainty introduced by different realizations of the same antenna during different SVs testing sessions by using a statistical representation in terms of expected pattern values and bounds.

**Figure 2:**
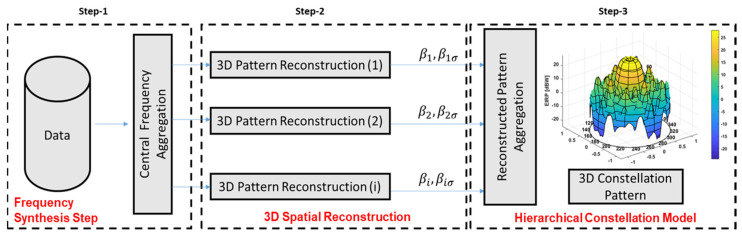
Antenna Pattern Multi−step Reconstruction Procedure.

The GRAP synthesis procedure has been split into three different steps. In the first step (Step-1 in Figure 2), a Power Spectral Density (PSD) Weighting is applied to the CW antenna measurements in order to resample the power distribution of the modulated signal in the target frequency band:

(2) $$G_{f}^{i}\left(\left.\theta ,\varphi \right|\theta_{1},\varphi_{1},f_{1},\theta_{2},\varphi_{1},\ldots \theta_{j},\varphi_{k}\right)$$

A different GRAP model can then be derived for the selected Galileo central frequencies considering f={E1,E5a,E5b,E6} [8]. Step-2 (Figure 2 ) introduces an improved 3D spatial model reconstruction to overcome scanning facility limitations and obtain an estimation of a smooth model (m) of the *i*-th antenna pattern $G_{f,m}^{i}\left(\theta ,\varphi \right)$ working closer to the expected physical behavior. Such an estimation can be obtained together with corresponding information about pattern spatial uncertainty, expressed by its model variance distribution (σ), which we indicate as $G_{f,\sigma }^{i}\left(\theta ,\varphi \right)$. The 3D patterns obtained are then used to feed the reference GRAP constellation model for a target central frequency $G_{f,m}^{R}\left(\theta ,\varphi \right)$ considering a hierarchical approach [18]. Specifically, the different radiation pattern realizations $G_{f,m}^{i}\left(\theta ,\varphi \right)$ relying on a specific space vehicle or test campaign session are processed as observations nested within different subjects at the bottom level, and belonging to the same constellation population at the top level. This defines a regression model allowing generalization of the antenna behavior (features) on top of its specific subject (single antenna patterns) or within subject realizations (measurements for different cuts and angles). The resulting $G_{f,m}^{R}\left(\theta ,\varphi \right)$ allows extension of the model to a more general representation compatible with possible realizations of the pattern family. The residual errors are still considered through estimation of the corresponding variance distribution $G_{f,\sigma }^{R}\left(\theta ,\varphi \right)$. The aggregation procedure can be generally described by (3).

(3) $$\left\{G_{f,m}^{1}\left(\theta ,\varphi \right),G_{f,m}^{2}\left(\theta ,\varphi \right),\ldots ,G_{f,m}^{n}\left(\theta ,\varphi \right),G_{f,\sigma }^{1}\left(\theta ,\varphi \right),G_{f,\sigma }^{2}\left(\theta ,\varphi \right),\ldots ,G_{f,\sigma }^{n}\left(\theta ,\varphi \right)\right\}\to \left\{G_{f,m}^{R}\left(\theta ,\varphi \right),G_{f,\sigma }^{R}\left(\theta ,\varphi \right)\right\}$$

Such a procedure corresponds to a specific statistical process, the mathematical implementation of which is described in the next sections.

### 2.1. Frequency Synthesis Step

The M available radiated field linear normalized power CW measurements at elevation and azimuth scanning points $\theta_{j},\varphi_{k}\;$ are combined as per the following:

(4) $${\left.G_{f}^{i}\right|}_{\theta_{j},\varphi_{k}}=\sum_{m=1}^{M}W_{{∆f}_{m}}^{PSD}{G(\theta }_{j},\varphi_{k})$$

The normalized weighting $W_{{∆f}_{n}}^{PSD}\;$ corresponds to the PSD of the E5a, E5b, and E6-B/C signals provided at intervals ∆*f* of 2.5 MHz. Uniform weighting is applied for the E1-B/C signal given the narrowband nature of this signal compared to the CW sampling interval. 

**Figure 3:**
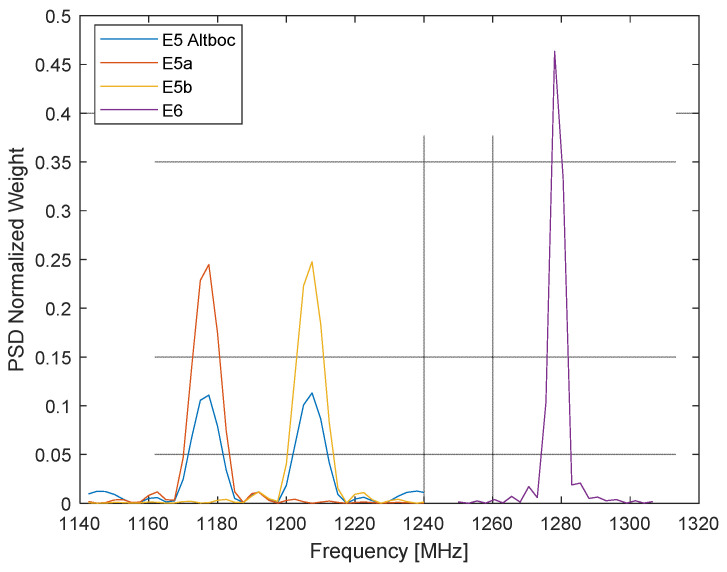
Distribution of $W_{{∆f}_{n}}^{PSD}$ for the different Galileo central frequencies.

The distribution of $W_{{∆f}_{n}}^{PSD}\;$ is represented with respect to frequency in Figure 3. The E5AltBOC combination is not considered hereafter, since the processing of the E5a and E5b patterns have been carried out separately [8]. The procedure moves the different CW samples to the target discrete spatial distribution ${\left.G_{f}^{i}\right|}_{\theta_{j},\varphi_{k}}$, that can then be used to feed the reconstruction procedure to obtain the specific subject $G_{f,m}^{i}\left(\theta ,\varphi \right)$ pattern realization for the selected frequency f.

### 2.2. A 3D Optimal Spherical Harmonic Based Spatial Reconstruction

The antenna spherical field characterization generally leads to a complex analysis, the results of which can also be far from the most effective realization. The common approach is to characterize the pattern by using a proper spherical harmonic expansion. As per [9,11] and [12,13], the reconstructed pattern can be expressed by using the following spherical harmonic $SH\left(\theta ,\varphi \right)\;$ expansion up to the order *N*

(5) $$SH\left(\theta ,\varphi \right)=\sum_{l=0}^{N}\sum_{m=-l}^{l}Y_{lm}\left(\theta ,\varphi \right)\beta_{lm}$$

with

(6) $$Y_{lm}\left(\theta ,\varphi \right)=\left\{\begin{array}{c} {\left(-1\right)}^{m}\sqrt{2}\sqrt{\frac{2l+1}{4\pi }\frac{\left(l-\left|m\right|\right)!}{\left(l+\left|m\right|\right)!}}P_{l}^{\left|m\right|}\left(cos\theta \right)\sin\left(\left|m\right|\varphi \right)\;if\;m<0\\ \sqrt{\frac{2l+1}{4\pi }}P_{l}^{\left|m\right|}\left(cos\theta \right)\;if\;m=0\\ {\left(-1\right)}^{m}\sqrt{2}\sqrt{\frac{2l+1}{4\pi }\frac{\left(l-m\right)!}{\left(l+m\right)!}}P_{l}^{m}\left(cos\theta \right)\cos\left(\left|m\right|\varphi \right)\;if\;m<0 \end{array}\right.$$

where $\beta_{lm}$ indicates the ${\left(N+1\right)}^{2}$ expansion coefficients and $P_{l}^{m}$ are the corresponding Legendre polynomials. The reconstruction problem can then be expressed in matrix form as

(7) $$G_{j,k}^{i}=Y\left(\theta_{j},\varphi_{k}\right)\beta^{i}+\nu_{j,k}^{i}$$

where Y is the spherical harmonic matrix operator computed by evaluating the spherical harmonic functions defined in (6) in the available observation points. The value $G_{j,k}^{i}\;$ indicates the *i*-th antenna measurements ${\left.G_{f}^{i}\right|}_{\theta_{j},\varphi_{k}}$ with spatial error $\nu_{j,k}^{i}$.

The standard least square solution can be poorly conditioned as far as higher harmonic order *N* is considered. Popular regularization strategies and basis pursuit favors physically admissible spherical fields with smooth variations, and allows better exploitation of the inner sparsity of the selected spherical harmonic feature space [9]. The application of a Sparse Estimator can select the relevant antenna model components [11] according to the data sampling and the level of noise. This work considers an elastic-net [15] variant, also proposed in [16], hence the target pattern parameter set can be computed as

(8) $$\hat{\beta_{i}}=\underset{\beta }{\min}\sum_{j=1}^{n}{\left\{G_{j,k}^{i}-Y^{*}\left(\theta_{j},\varphi_{k}\right)\beta_{i}\right\}}^{T}\left\{G_{j,k}^{i}-Y^{*}\left(\theta_{j},\varphi_{k}\right)\beta_{i}\right\}+n\lambda \left[{(1-\alpha )\left\|\beta_{i}\right\|}_{2}^{2}+{\alpha \left\|\beta_{i}\right\|}_{1}\right]$$

where the loss function has been slightly modified considering

(9) $$Y^{*}\left(\theta_{j},\varphi_{k}\right)=\Gamma^{-1/2}Y\left(\theta_{j},\varphi_{k}\right)$$

where Γ is a weighting variance matrix iteratively estimated from the regression residuals in order to approximate the normalized linear pattern log-normal distribution [19]. 

**Figure 4:**
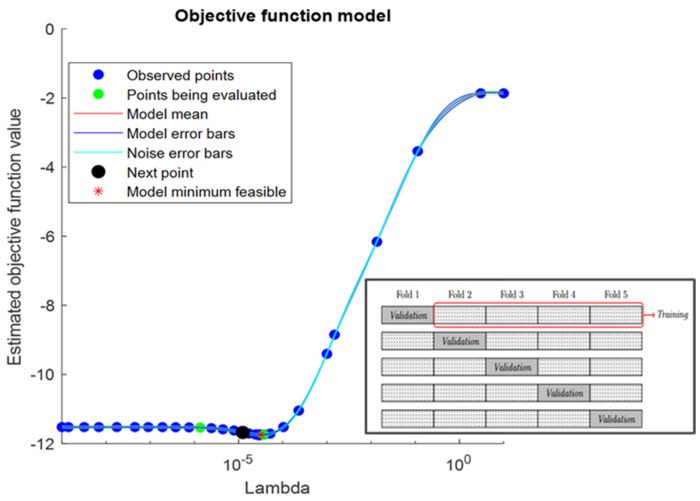
Elastic Net Learning curve for E1 $\lambda_{min}$ determination.

A balance between smoothing and shrinkage has been considered (α = 0.5), whereas the λ key hyper-parameter has been found by using a Bayesian minimization of the *k*-fold cross-validation error (with *k* = 10) [15]. The cross-validation splits the dataset of the pattern into training and validation subsets. The optimal lambda $\lambda_{min}$ will be one that minimize the loss function computed by solving the regression on the training set and evaluating model predictions against the validation data. For the antenna pattern problem, this corresponds to learning the harmonic content of a subset of data and optimizing the capability of the model to predict another validation section (i.e., angular sector). The maximum order considered for $SH\left(\theta ,\varphi \right)$ is N=60, so the maximum feature space $\hat{\beta_{i}}$ considers 3721 components. The hyper-parameter is derived once per pattern family, i.e., E1 frequency patterns, and then included to solve (8). The learning curve for the E1 frequency pattern family is shown in Figure 4: the process achieves a minimum $\lambda_{min}$ for which the model does not learn much more by adding non-zero $\beta_{lm}$ coefficients. The curve can be assumed as a reference example of the optimization process for the other frequency cases E5a,E5b,E6 as well, which converge to their specific hyper-parameter values.

The target regressor problem in (8) aims at estimating not only the reconstruction coefficient $\hat{\beta_{i}}$, providing the 3D reconstruction:

(10) $$G_{f,m}^{i}\left(\theta ,\varphi \right)=Y\left(\theta ,\varphi \right)\hat{\beta_{i}}$$

but also the associated covariance matrix $\Sigma_{\hat{\beta_{i}}}$ allowing approximation of the expected spatial uncertainty in different pattern regions by using the SH operator:

(11) $$G_{f,\sigma }^{i}\left(\theta ,\varphi \right)=Y\left(\theta ,\varphi \right)\Sigma_{\hat{\beta_{i}}}{Y\left(\theta ,\varphi \right)}^{T}$$

A bootstrap approach [15] has been considered here to better estimate $\Sigma_{\hat{\beta_{i}}}$ for the regressor exploited in (8). Residual resampling performed one hundred times is sufficient to converge to a good approximation, in line with the small deviation experienced from the data.

### 2.3. Hierarchical Constellation Model

The constellation pattern hierarchical model considers two representation layers:

- The individual models of the specific antenna pattern $G_{f,m}^{i}\left(\theta ,\varphi \right)$.
- The population average model or the constellation pattern $G_{f,m}^{R}\left(\theta ,\varphi \right)$.

The method of obtaining the individual estimate of $G_{f,m\;}^{i}$ is the reconstruction procedure defined in the previous section, with the possibility of mapping the problem from the spatial domain to the parameter space $\beta_{i}$. In such a space, the population model can be expressed by a simplified normally distributed population $\beta_{i}\sim N\left(\beta_{\mu },\Sigma_{\beta_{i}}\right)$, where individual specific realizations are expressed as

(12) $$\beta_{i}=\beta_{\mu }+b_{i}\;E(b_{i})=\mu_{b}=0\;Var\left(b_{i}\right)=D$$

which is a linear combination of the population *grand mean* coefficients $\beta_{\mu }$ and $b_{i}$, referred to as between-subject random effects [18]. D defines the between-subject associated covariance, and $\mu_{b}$ is their mean. Considering that the first reconstruction step provides only an estimate of parameters, (12) can be rewritten as

(13) $$\hat{\beta_{i}}\approx \beta_{\mu }+b_{i}+\varepsilon_{i}^{*}\;Var\left(\varepsilon_{i}^{*}\right)=\Sigma_{\hat{\beta_{i}}}$$

where $\varepsilon_{i}^{*}$ is the reconstruction error and $\Sigma_{\hat{\beta_{i}}}$ is the uncertainty associated with the reconstruction procedure for the subject *i*. Equation (13) allows us to estimate the population average parameters $\beta_{\mu }$ by the following weighted least square solution:

(14) $$\hat{\beta_{\mu }}={\left({Ι}^{T}{\left(V\right)}^{-1}I\right)}^{-1}{Ι}^{T}{\left(V\right)}^{-1}\hat{\beta_{1,2,\ldots L}}\Sigma_{\beta_{\mu }}={\left({Ι}^{T}{\left(V\right)}^{-1}I\right)}^{-1}$$

Here, $\hat{\beta_{1,2,\ldots L}}$ is the concatenation of dimension LNx1 of vector parameters estimated for all antenna patterns 1,2,…L, I is a block diagonal matrix with unit matrix blocks $1_{NxN}$**,** and V is the block diagonal matrix with element $V_{i}=D+\Sigma_{\hat{\beta_{i}}}$. This solution assumes that covariances D and $\Sigma_{\hat{\beta_{i}}}$ are known. $\Sigma_{\hat{\beta_{i}}}$ is provided by the first regression step, whereas D is computed according to the sample variance estimator:

(15) $$\frac{\Delta \beta_{i}\Delta {\beta_{j}}^{T}}{N-1}={\left(N-1\right)}^{-1}\sum_{i=1}^{N}{\left(\hat{\beta_{i}}-\hat{\beta_{\mu }}\right)\left(\hat{\beta_{j}}-\hat{\beta_{\mu }}\right)}^{T}\;\;i\neq j$$

and then refined by recursive iterations. Once the population mean $\hat{\beta_{\mu }}$ and its asymptotic covariance $\Sigma_{\hat{\beta_{\mu }}}$ are determined, it is possible to define the final population model by averaging (13) with respect to single subject realization, hence:

(16) $$E\left(\hat{\beta_{i}}\right)=\hat{\beta_{\mu }}\;\;\;Var\left(\hat{\beta_{i}}\right)=\Sigma_{\hat{\beta_{\mu }}}+D+\Sigma_{\hat{\beta_{i}}}$$

with $\Sigma_{\hat{\beta }}={\left(N\right)}^{-1}\sum_{i=1}^{N}\Sigma_{\hat{\beta_{i}}}$. The constellation model GRAP can be finally defined as per Section 2.2, in terms of its mean $G_{m,\sigma }^{R}\left(\theta ,\varphi \right)$ and variance $G_{f,\sigma }^{R}\left(\theta ,\varphi \right)$ by simply mapping equation (16) through a spherical harmonic operator:

(17) $$\begin{array}{c} G_{f,m}^{R}\left(\theta ,\varphi \right)=Y\left(\theta ,\varphi \right)\hat{\beta_{\mu }}\\ G_{f,\sigma }^{R}\left(\theta ,\varphi \right)=Y\left(\theta ,\varphi \right)\left(\Sigma_{\hat{\beta_{\mu }}}+D+\Sigma_{\hat{\beta }}\right){Y\left(\theta ,\varphi \right)}^{T} \end{array}$$

## 3. Model Derivation Results 

In this section, the GRAP for the E1 frequency is analyzed with respect to the implemented procedure. 

**Figure 5:**
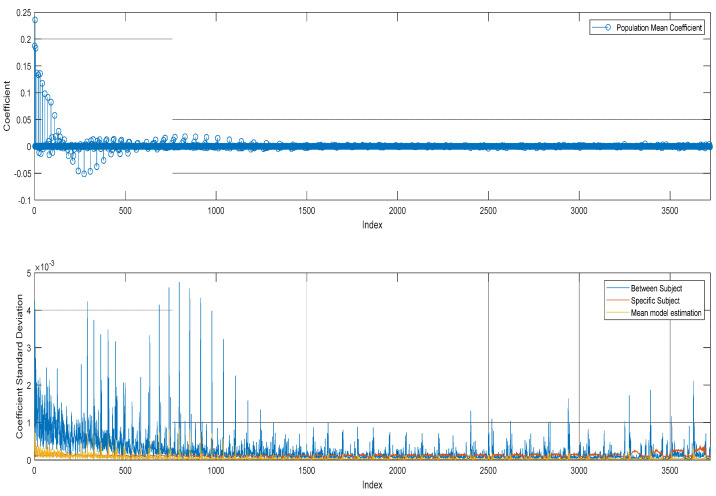
Spherical Harmonic Parameter Space representation of the GRAP pattern: upper—E1 GRAP coefficients $\hat{\beta_{\mu }}$, bottom—corresponding standard deviations.

The representation in the parameter space is provided in Figure 5. The upper plot provides the E1 GRAP coefficients $\hat{\beta_{\mu }}$, confirming the good capability of the regression process to emphasize or shrink toward zero the different harmonic components according to their relevance. The bottom plot shows the different covariance contributions defined in (16) $\Sigma_{\hat{\beta_{\mu }}},D,\Sigma_{\hat{\beta }}$ in terms of corresponding standard deviations in the coefficient domain. The three contributions respectively represent the mean model estimation variance (yellow), the between-subject variance (blue), and the specific subject mean variance (red). The proposed hierarchical aggregation in the parameter space allows us to isolate the principal antenna pattern components $\hat{\beta_{\mu }}$ from the realizations of specific testing campaigns or physical antenna detours, and move them into different variance contributions. This is in line with objective (3) defined in Section 2. 

The Y operator allows us to generate the $G_{f,m}^{R}\left(\theta ,\varphi \right)$ and $G_{f,\sigma }^{R}\left(\theta ,\varphi \right)$ for a target point $\left(\theta ,\varphi \right)$ according to (17). This possibility fits with objective (2) in Section 2, hence the model can cope with improved resolution with respect to that provided by measured samples.

The reconstructed GRAP information has been made available to the final user in [8], evaluating the pattern function on a 1 deg resolution grid θxφ = [0, 90] × [0, 360], which simultaneously guarantees enough accuracy via standard interpolation methods and provides an efficient lookup table for user implementation. 

According to the reference document [8], the GRAP metadata are represented on such a grid in terms of expected Equivalent Isotropic Radiated Power (EIRP) variation with respect to the azimuth and co-elevation angles. This representation can be easily derived from the estimated pattern $G_{m,\sigma }^{R}\left(\theta ,\varphi \right)$, considering

(18) $${EIRP}_{f,GRAP,dBW}^{R}\left(\theta ,\varphi \right)={Pt}_{f,dBW}+G_{f,m,dB}^{R}\left(\theta ,\varphi \right)$$

where $G_{f,m,dB}^{R}\left(\theta ,\varphi \right)$ is the dB conversion of $G_{f,m}^{R}\left(\theta ,\varphi \right)$ considering the transformation provided in Appendix A, and ${Pt}_{f,dBW}$ is the in-band transmitted power. As per [8], the considered ${Pt}_{f,dBW}$ offset has been computed in order to ensure that the minimum user received power at ground level does correspond to the Galileo commitments for the different signals E5a, E5b, E6, and E1 defined in [20]. 

**Figure 6:**
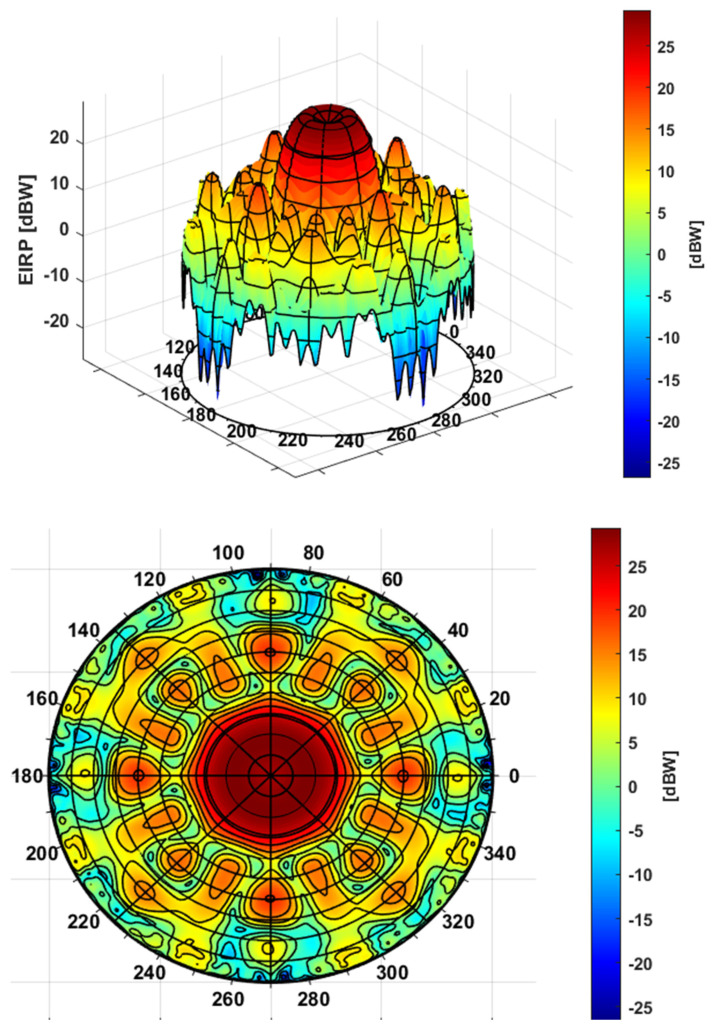
Galileo E1 ${EIRP}_{E1,m,dBW}^{R}\left(\theta ,\varphi \right)$ 3D representation (**top**) and correspondent polar plot (**bottom**).

The final 3D plot of the ${EIRP}_{E1,m,dBW}^{R}\left(\theta ,\varphi \right)$ and the corresponding polar plot (i.e., with respect to azimuth and radial component) are provided in Figure 6. Polar plot distribution is particularly relevant since it shows that the final user information considers the azimuth asymmetries of the antenna pattern. Those details can be considered as constellation antenna design specific features emerging from the different realizations.

One of the most relevant properties of the reconstruction based on spherical harmonic expansion is to filter out spatial discontinuities and noise effect in the data, moving the distribution closer to its expected physical behavior. This can be appreciated computing the 2D Laplacian of the averaged raw spherical scanning distribution for the target E1 frequency with respect to the developed 3D reconstructed pattern.

**Figure 7:**
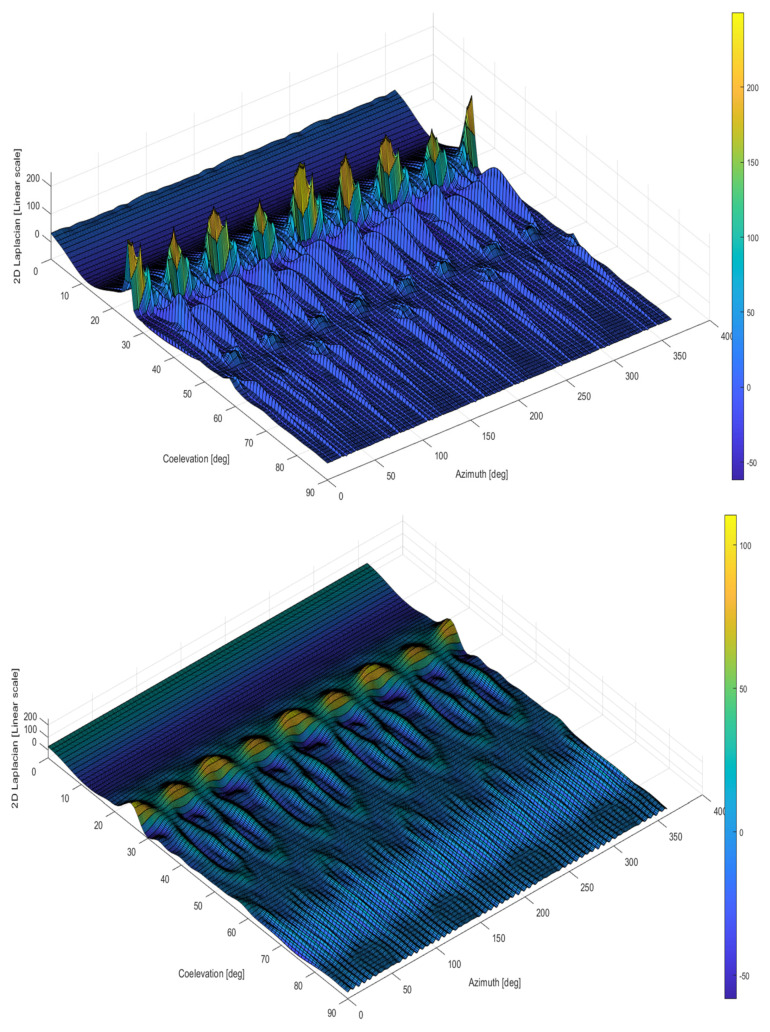
Galileo E1 Laplacian for data sample mean (**top**) and reconstructed pattern (**bottom**).

Such a comparison is provided in Figure 7, where upper and lower distributions represent 2D Laplacian applied to the raw data sample mean and the reconstructed pattern, respectively. In the latter case, it can be appreciated that the secondary lobes variation will follow a smooth transition from one angular position to the other. This completes GRAP model objective (2) by providing the advantage on the user side of mitigating unexpected discontinuities during GNSS receiver tracking simulations.

According to (17), from the parameter space reconstruction it is also possible to reconstruct the pattern variance $G_{f,\sigma }^{R}\left(\theta ,\varphi \right)$. As per [8], this information can be effectively exploited to define a model confidence bound, which is expressed as user EIRP to represent 95% of the expected variation.

Therefore, the upper ${\left.{EIRP}_{CI,f,dBW}\left(\theta ,\varphi \right)\right|}_{+}$ and lower ${\left.{EIRP}_{CI,f,dBW}\left(\theta ,\varphi \right)\right|}_{\left(-\right)}$ bounds have been defined by using the following expressions:

(19) $${\left.{EIRP}_{CI,f,dBW}\left(\theta ,\varphi \right)\right|}_{\left(+/-\right)}={EIRP}_{f,GRAP,dBW}^{R}\left(\theta ,\varphi \right)+{2\sigma }_{f,dB}^{R}\left(\theta ,\varphi \right)$$

Here, $\sigma_{f,dB}^{R}\left(\theta ,\varphi \right)$ is derived from the variance matrix $G_{f,\sigma }^{R}\left(\theta ,\varphi \right)$ and converted into dB according to the formulas provided in Appendix A.

**Figure 8:**
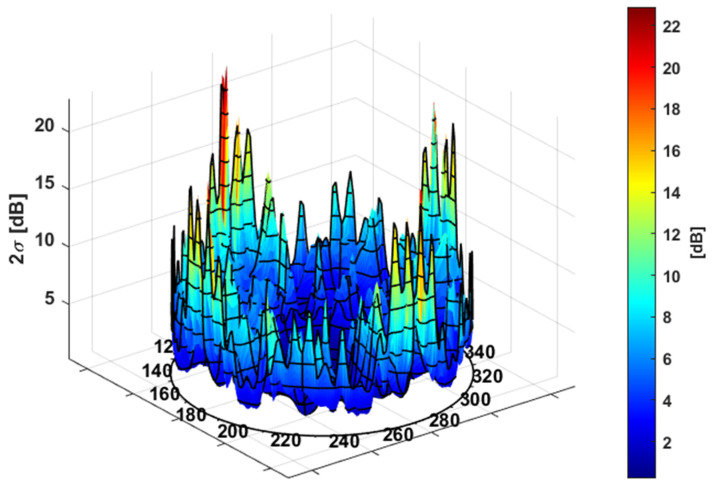
E1 3D Constellation expected 95% variation: ${2\sigma }_{f,dB}^{R}\left(\theta ,\varphi \right)$.

Figure 8 shows the final 3D representation of the estimated uncertainty ${2\sigma }_{f,dB}^{R}\left(\theta ,\varphi \right)$. As expected, the uncertainty radially increases towards the extreme ends of the distribution and preserves azimuth diversity due to antenna-specific features.

**Figure 9:**
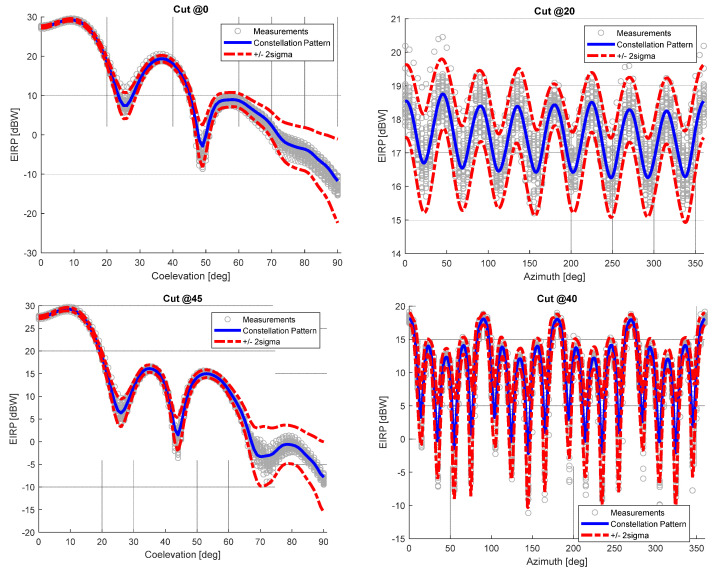

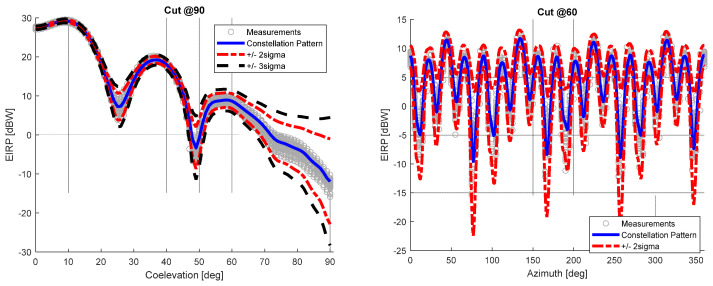
Galileo E1 Constellation pattern at different co-elevation and azimuth cuts (blue line) together with confidence bounds (red dotted line) vs measurement data (grey samples).

Evidence that the defined bounds are able to asymptotically represent the data excursion is provided in Figure 9. It can be recognized as a good match with the measurement samples, which fall within the upper and lower bounds for different co-elevation and azimuth cuts.

Figure 9 also provides in the bottom left plot the$\;{3\sigma }_{f,dB}^{R}\left(\theta ,\varphi \right)$ bound in order to appreciate that the model can be generally used to extend the statistical test coverage. The bound slightly overestimates the variability at higher off-boresight angles (θ> 60), reflecting the complexity of predicting the pattern behavior in the regions that experience very small values and high spatial variation. However, the effect applies only to very small EIRP values, so in terms of power distribution its effect has been considered acceptable, also taking into account that it provides a conservative estimation. It is worth noting that the work performed on the definition of bounds is mainly devoted to complementing the model with compact information for the user about the expected excursion in a specific angular region.

**Figure 10:**
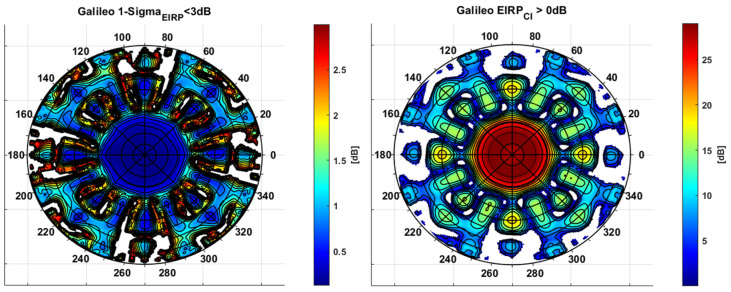
E1 Galileo GRAP stability and strength for SSV applications.

In Figure 10 (left-hand side), the polar plot for $\sigma_{f,dB}^{R}\left(\theta ,\varphi \right)<3dB$ is provided. A few areas, mainly related to the highest off-boresight angles and around nulls, do not cope with this requirement (white areas), confirming the expected high stability of Galileo FOC constellation patterns across the different realizations. Figure 10 (right-hand side) also provides ${\left.{EIRP}_{CI,f,dBW}\left(\theta ,\varphi \right)\right|}_{\left(-\right)}>0dB$, which can be considered representative of the Galileo E1 EIRP strength. It can be seen that Galileo can guarantee a minimum positive EIRP up to 55 degrees off boresight angles (about 0.6 radii in polar plot), excluding a few localized regions (white areas).

Those kinds of features can be further investigated by using the GRAP metadata provided in [8] for the other Galileo signal carrier frequencies. In the next section, a simplified SSV geometrical analysis is introduced to better connect the GRAP to the final Galileo SSV link budgets and address its exploitation for a target space user.

## 4. Galileo Pattern Driven Space Service Volume Analysis

### 4.1. SSV Geometrical Model and Accessibility Index Definition

The Galileo Antenna Reference Pattern information can be adopted for mission feasibility analysis and spaceborne receiver design. This section proposes a first-order analytical procedure allowing preliminary characterization, with respect to a target mission altitude, of the effect of the GRAP EIRP on the expected SSV performance.

**Figure 11:**
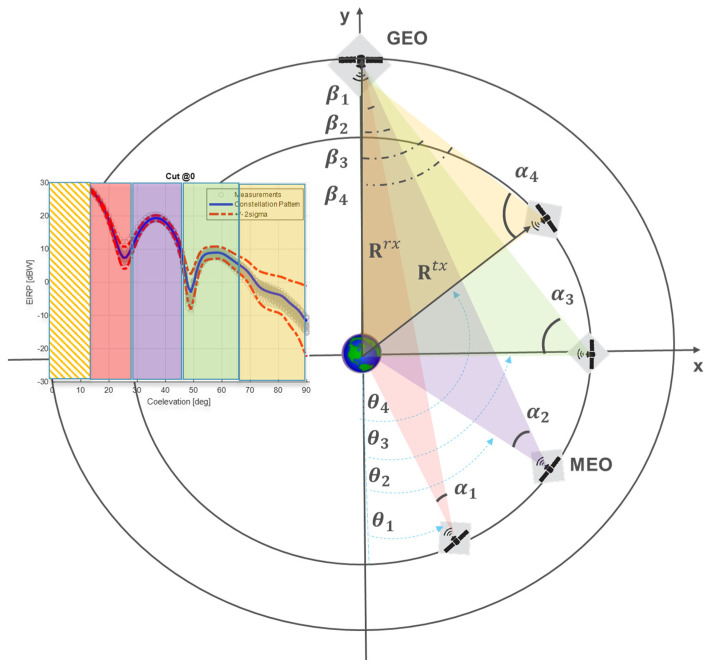
Space Service Volume simplified geometry for accessibility index definition.

The approach is based on a simplified service geometry represented in Figure 11.

Let us consider:

- A target spacecraft *rx* receiving the Galileo signal and crossing the plane defined by the orbital plane of a reference *tx* Galileo satellite. Such an event defines a simplified geometry condition where the *rx* user can be assumed at fixed position $R^{rx}$ and the transmitting Galileo is located at its rising position $R^{tx}$ along its orbit.
- An *rx* Earth-centred 2D reference frame lying in the Galileo orbital plane and considering y-axis aligned to ${\hat{R}}^{rx}$. With this assumption, the 2D *rx* position vector is $\;R^{rx}=\left\{0,r_{y}\right\}$ and depends on the user altitude considering only $r_{y}=r_{Earth}+h_{rx}$. In Figure 11, a GEO satellite use case is shown, so $r_{y}$ would be $r_{Earth}+h_{GEO}$.
- Different crossing events *i* can be defined so far as the transmitting MEO rising position can be placed at any point in its orbit (circularly approximated). This is represented by different MEO SV positions drawn in Figure 11. Such a sequence of Galileo *tx* positions can be defined in the RXRF reference frame as $R_{i}^{tx}=\{r_{x,i}^{tx},r_{y,i}^{tx}\}$ with $\left|R_{i}^{tx}\right|=r_{Earth}+h_{MEO}$. It is assumed $\hat{R_{i}^{tx}}\equiv -\hat{n_{i}}$, where $n_{i}$ is the transmitting antenna boresight nadir-pointing direction. Attitude corrections, as per yaw steering, are outside the scope of the simplified geometry.
- The symmetry of the problem allows us to consider the subset of events where $r_{x,i}^{tx}>0$.

The proposed geometry focuses on the upper SSV layer, where target space vehicle altitudes are higher than the MEO constellation ones, so it is assumed $h_{rx}>h_{tx}$. This is in line with the standard expected SSV receiving conditions, considering receiver antennas pointing toward the Earth and the GNSS signals generated by satellites rising above the Earth disk. Now, consider the correspondingly defined angles:

$\theta_{i}$ the anomaly angle sequence spanned by $R_{i}^{tx}\;$ with $\theta_{i}\in \left[0,\pi \right]$, stated $r_{x,i}^{tx}>0$;

$\alpha_{i}$ angle defined by $R^{tx}$ and $R^{rx}$ according to the geometrical relation

(20) $$\alpha_{i}{=\cos}^{-1}\frac{{\left|R^{tx}\right|}^{2}+{\left|R^{tx-rx}\right|}^{2}-{\left|R^{rx}\right|}^{2}}{2\left|R^{tx}\right|\left|R^{tx-rx}\right|}$$

where $R^{tx-rx}=R^{tx}-R^{rx}$. The proposed domain $\left\{\alpha_{i},\theta_{i}\right\}$ is particularly suitable for linking the pattern region spanned by different relative rx and tx positions considering that each $\alpha_{i}$ corresponds to the antenna pattern off-boresight angles experienced by the GNSS SV for a target transmitting position $\theta_{i}$ (Figure 11, left side pattern representation). It is clear that, according to the altitude of the user $h_{rx}$, the sequence $\theta_{i}$ of a rising satellite can cover the whole antenna pattern off-boresight range [0, π/2] without spanning the whole $\theta_{i}\;$ interval [0,π]. Therefore, it is possible to define that a rising transmitter position θ can be considered compatible only for θ : α≤π/2. It is expected that as the altitude increases from GEO to HEO (and above) the θ compatible interval shrinks according to (20). The interval $\theta_{i}$ is also restricted to [$\theta_{Earth}$,π] considering the Earth shadowing angle provided by $\theta_{Earth}=\sin^{-1}\left(\left|R_{Earth}\right|/\left|R^{rx}\right|\right)$. 

**Figure 12:**
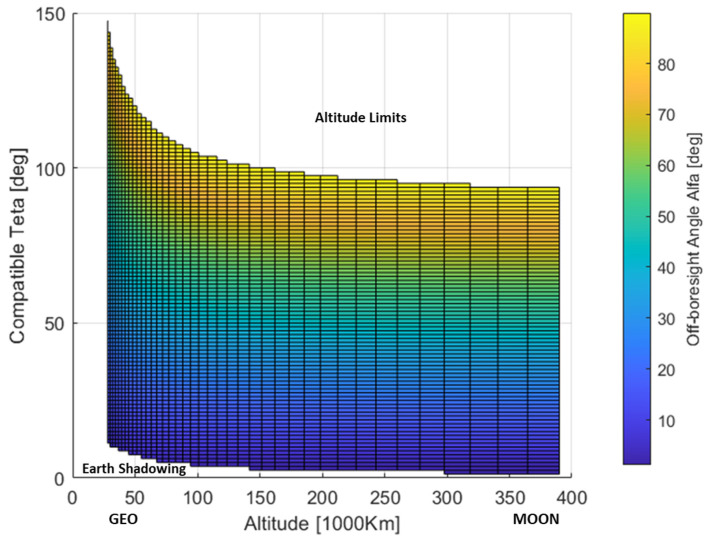
Off- boresight angle antenna pattern spanned for different SV rising positions and user altitudes.

The compatible set [$\theta_{Earth}$,$\;\theta_{\alpha =\pi /2,\;h=h_{rx}}$] can be intended as a measure of the geometrical compatibility of the Galileo transmitting positions with respect to the SSV end-user. Figure 12 represents the distribution $\alpha_{i}\left(\theta_{i},h_{rx}\right)\;$ of the spanned off-boresight angles on a 2D grid. The $\theta_{i}$ step size is 1.25 deg and ${\;h}_{rx}$ is considered equally spaced in log-scale and spanning the interval $\left[28\times 10^{6},\;390\times 10^{6}\right]$ meters, which covers SSV altitudes up to lunar orbits. This $\left\{\theta_{i},h_{rx}\right\}$ grid will be assumed as a reference to represent all the distributions hereafter considered. In Figure 12, the two competitive effects affecting the $\theta_{i}$ set are clearly highlighted as far as the altitude increase: (1) the reduction of the Earth disk obscuration; (2) the reduction of maximum compatible $\theta_{i}$ corresponding to the maximum $\alpha_{i}=\pi /2$.

Considering this domain $\left\{\theta_{i},h_{rx}\right\}$, the SSV Received Signal Power $C_{SSV}^{R}$ can be easily defined to map the experienced off-boresight angles through the GRAP EIRP model and considering the correspondent path loss [21]:

(21) $$C_{SSV}^{R}\left(\theta_{i},\varphi ,h_{rx}\right)={EIRP}_{\varphi *}^{R}\left(\alpha_{i},\varphi \right)-20log\left(\frac{4\pi \left|R_{h_{rx}}^{tx-rx}\right|}{\lambda }\right)$$

where λ is the E1 carrier signal wavelength, and (21) is intended as a function of the receiver altitude $h_{rx}$ through $R_{h_{rx}}^{tx-rx}$ and $\theta_{i}$ according to the representation defined. Equation (21) can be used to express the system performance for different EIRP azimuth angles spanning φ∈[0,2π]. In addition, assuming that for the simplified geometry, different φ can be equivalently experienced, it is possible to build up synthetic indexes like:

(22) $$\begin{array}{c} C_{SSV,min}^{R}\left(\theta_{i},h_{rx}\right)=\underset{\varphi \in [0,2\pi ]}{\min}C^{R}\left(\theta_{i},\varphi ,h_{rx}\right)\\ C_{SSV,ave}^{R}\left(\theta_{i},h_{rx}\right)=\frac{1}{2\pi }\sum_{\varphi \in [0,2\pi ]}C^{R}\left(\theta_{i},\varphi ,h_{rx}\right) \end{array}$$

which define the worst case and average SSV received signal power, respectively. The minimum and average are computed considering the 1 deg resolution discrete grid defined for φ∈[0,2π]. The so-defined $C_{SSV}^{R}\left(\theta_{i},\varphi ,h_{rx}\right)$ can also be exploited to introduce a novel compact index, namely Galileo SSV accessibility. 

This index is intended as a measure of the fraction of the rising Galileo orbit arc that the user can potentially exploit to access an SV signal during a crossing event for a target minimum received signal power threshold $C_{Min}^{R}$. In fact, considering the crossing event as uniformly distributed with respect to the current position of the rising Galileo *tx*, such an indicator can be expressed as a percentage $A_{\%}$, considering the ratio between the effective arc $\theta_{eff}$ (spanning signal power values ${C^{R}>C}_{Min}^{R}$) and the maximum allowed arc $\theta_{tot}$

(23) $${\left.A_{\%}\right|}_{C}=\frac{{\left.\theta_{eff}\right|}_{{C^{R}>C}_{Min}^{R}}}{\theta_{tot}}=\frac{{\left.\theta_{eff}\right|}_{{C^{R}>C}_{Min}^{R}}}{\pi }\;{\;with\;\;\theta }_{eff}(h_{rx})=\theta_{max}-\theta_{min\;}\;$$

where $\theta_{max}$ and $\theta_{min\;}\;$are respectively the maximum angle $\theta_{i}$ satisfying ${C^{R}>C}_{Min}^{R}$, and the minimum $\theta_{i}$ geometrically compatible at altitude $h_{rx}$. $\theta_{tot}=\pi$ can be easily retrieved by the geometry considered herein (Figure 11). For $\theta_{max}=\theta_{\alpha =\pi /2}$, the Equation (23) defines the geometrical accessibility, hence one that can be achieved removing the limitation on the minimum received signal power.

Moving from service to user side, the introduction of a high-gain antenna is generally considered in order to leverage SSV navigation limits. Assuming the geometry in Figure 11, and a receiving antenna pattern equally distributed in azimuth, the receiving antenna gain can be evaluated by

(24) $$G_{Rx}\left(\beta_{i}\right)\;\mathrm{with}\;\beta_{i}{=\cos}^{-1}\frac{{\left|R^{rx}\right|}^{2}+{\left|R^{tx-rx}\right|}^{2}-{\left|R^{tx}\right|}^{2}}{2\left|R^{tx}\right|\left|R^{tx-rx}\right|}$$

where $\beta_{i}\;$is the receiving antenna off-boresight angle corresponding to a transmitting position $\theta_{i}$. 

**Figure 13:**
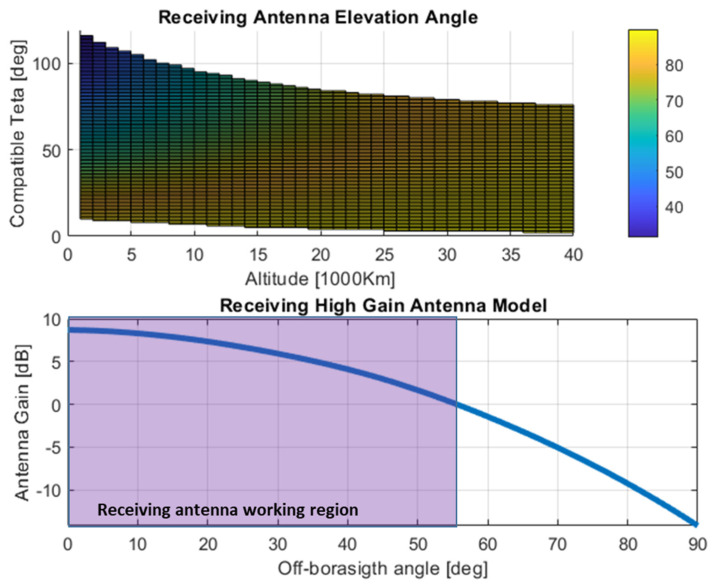
Receiving antenna off-boresight angles vs altitude and compatible teta (**top**) and receiving working region for reference high gain receiving antenna pattern (**bottom**).

As shown in Figure 13 (top), the usage of a high-gain antenna is also justified by considering that the spanned elevation $\frac{\pi }{2}-\beta_{i}$ converges to very high values as the altitude increases. The restricted receiving working region is shown in the bottom of Figure 13 for a representative high-gain antenna model, which will be considered as reference in the rest of the analysis.

It is now possible to introduce in the link budget assessment [21] the receiver sensitivity expressed in terms of carrier to noise ratio C/N0 [dB/Hz]. Such a value, without considering implementation losses [21], can be easily computed from the available signal carrier power $C^{R}\left(\theta_{i},\varphi ,h_{rx}\right)$, adding the amplification effect of $G_{Rx}\left(\beta_{i}\right)$ from (24) and subtracting the receiver equivalent thermal noise $N_{0}$

(25) $${C/N_{0}}_{SSV}^{R}\left(\theta_{i},\varphi ,h_{rx}\right)=C^{R}\left(\theta_{i},\varphi ,h_{rx}\right)+G_{Rx}\left({\left.\beta_{i}\right|}_{\alpha_{i}}\right)-10log10(KT_{Sys})$$

where K is the Boltzmann constant and $T_{Sys}$ is the equivalent system temperature in Kelvin [21]. From (25), synthetic performance indexes, i.e., expected average or worst-case conditions, can be derived as per (22).

Calculating the full link budget allows us to also express the accessibility in terms of receiver sensitivity, i.e., the fraction of the rising Galileo orbit arc providing a carrier-to-noise ratio above a predefined threshold ${\left.C/N_{0}\right|}_{Min}$:

(26) $${\left.A_{\%}\right|}_{C/N_{0}}=\frac{{\left.\theta_{eff}\right|}_{C/N_{0}>{\left.C/N_{0}\right|}_{Min}}}{\pi }{\;with\;\;\theta }_{eff}(h_{rx})=\theta_{max}-\theta_{min\;}$$

It is worth bearing in mind that $C/N_{0}$ accessibility based on (25) is intended as a simplified version of the link budget, which is sufficient for a first-order characterization of the SSV general performance. Starting from (25), specific user evaluation can customize the process by considering more complex signal propagation and implementation losses. The receiving antenna pattern can deviate from the assumed reference (Figure 13), and it can also be a function of the azimuth. Those limits do not jeopardize the generality of the results provided in the next section and the assessment of the expected E1 Galileo SSV volume peculiarities.

### 4.2. SSV Galileo E1 Characterization Results

The first significant variable to be considered is the SSV available signal power. 

**Figure 14:**
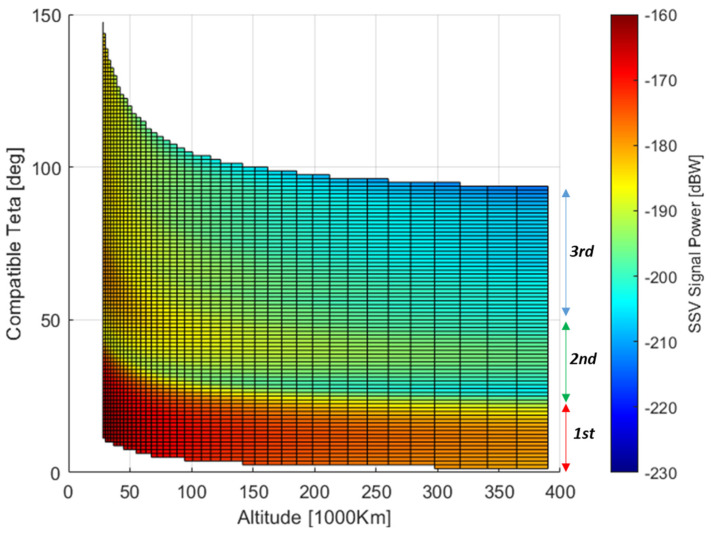
$\left(\theta_{i},h_{rx}\right)\;$received signal power distribution for Galileo E1.

**Figure 15:**
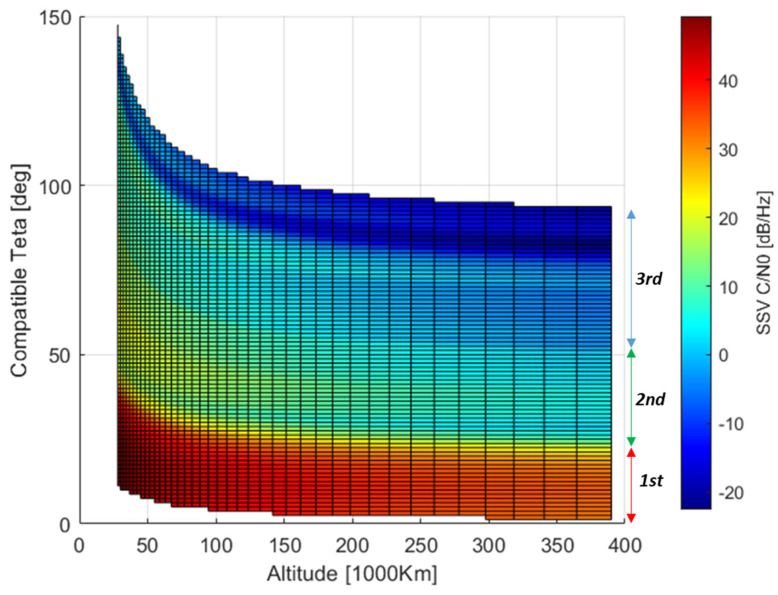
$\left(\theta_{i},h_{rx}\right)\;$carrier-to-noise ratio distribution for Galileo E1.

The average distribution expressed by (22) is represented in Figure 14. The effect of the GRAP pattern variation for high off-boresight angles (side-lobes) can be clearly indicated by correlating the spanned off-boresight angles in Figure 12 with the corresponding signal power in Figure 14. It is also possible to see that as the altitude increases, the system service converges into three main regions. They are identified by different levels of power, approximately $C_{SSV,ave}^{R}$ > 185 dBW, −205 dBW ${<C}_{SSV,ave}^{R}$ < −185 dBW, $C_{SSV,ave}^{R}$ < −205. According to (25), similar behavior on the user side is expected by computing ${C/N0}_{SSV,ave}^{R}\left(\theta_{i},h_{rx}\right)$. Such a distribution is provided in Figure 15, where the asymptotic separation of the three regions is even sharper.

The distributions provided can be easily expressed in terms of accessibility, which aggregates the information in a more compact index $A_{\%}$. Considering Equations (23) and (26) for different thresholds of the $C_{SSV,ave}^{R}\left(\theta_{i},h_{rx}\right)$ and ${C/N0}_{SSV,ave}^{R}\left(\theta_{i},h_{rx}\right)$, it is possible to define the average accessibility charts describing the expected improvements when moving from one threshold to the other.

**Figure 16:**
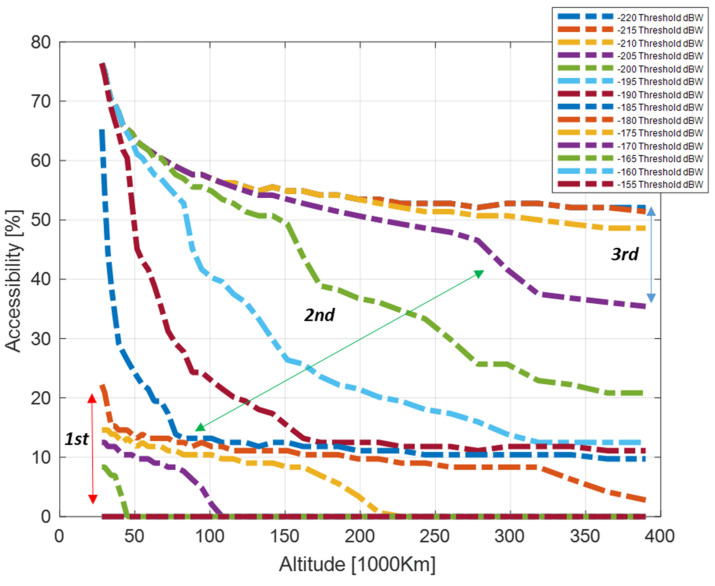
Accessibility chart for Galileo E1 for target received signal power.

**Figure 17:**
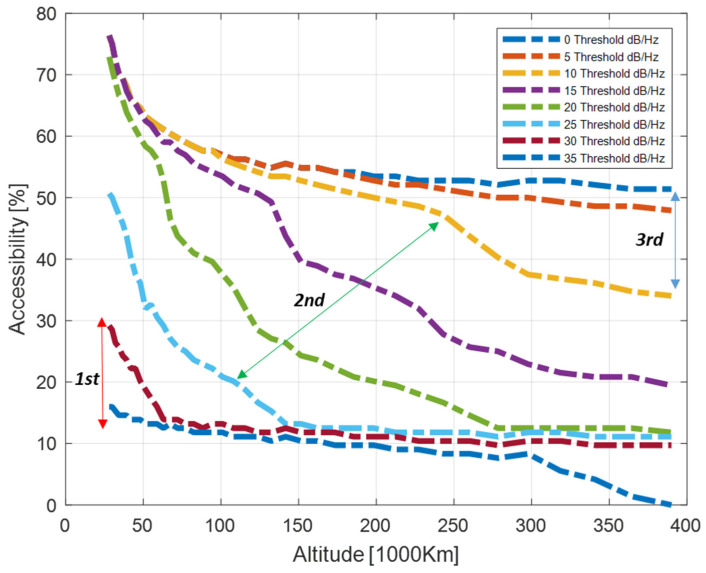
Accessibility chart for Galileo E1 for target receiver sensitivity (${C/N_{0}}_{min})$.

Those charts are represented in Figure 16 and Figure 17, respectively, considering the interval $C_{min}^{R}\in \left[-220,-155\right]\;$ with a step size of 5 dBW for the received signal power and an interval of ${C/N0}_{min}^{R}\in [0,\;35]$ with a step size of 5 dB/Hz for the carrier-to-noise ratio. The charts should be interpreted with respect to system and user requirements as follows. The system can guarantee more than 20% average accessibility up to 200 thousand kilometers for a user whose receiver design can cope with a received power level of −195 dBW (cyan curve in Figure 16); considering a fixed GNSS assembly (receiver plus receiving antenna), and a sensitivity of 25 dB/Hz (cyan curve in Figure 17), the user can benefit from at least 10% accessibility from HEO to the Moon.

It can be noted that the accessibility index possesses a great capability for representing the expected non-linear SSV performance variation. The second region, identifying side lobes access, is very well represented since the same signal power or receiver sensitivity increment corresponds to larger variations of the percentage, hence a larger increment of the effective Galileo orbit arc that can be exploited for navigation. It should also be noted that the sensitivity ranges 10–25 dB/Hz, accessing the second region at very high altitudes, is in line with current receiver solutions proposed for navigation in SSV up to lunar orbits [1].

Accessibility also has the advantage of aggregating the information in a scalar index, so it allows better visualization of E1 GRAP driven SSV with respect to azimuth variation. In fact, it is possible to compute (23) and (26) as functions of the azimuth through (21) and (25), hence providing azimuth-dependent accessibility distributions. 

**Figure 18:**
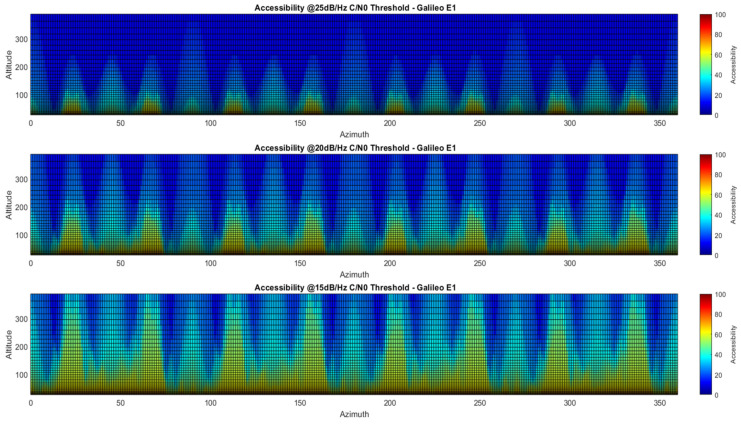
Variation with respect to azimuth for three different tracking thresholds: 15, 20, 25 dB/Hz.

For the sake of brevity, only ${\left.A_{\%}\right|}_{C/N_{0}}$ variation with respect to azimuth is provided in Figure 18.

As the sensitivity increases, the accessibility increases, but more importantly several azimuth intervals become active in the sense of accessing a longer Galileo satellite arc. In the azimuth-dependent accessibility plots (Figure 18), sixteen beacons can be clearly recognized, including eight which are much stronger than the rest. For more than 20 dB/Hz, such beacons support satisfactory accessibility up to lunar altitude. The azimuth-dependent results complement the average accessibility, revealing more details about azimuth intervals where service can outperform the average value. The two metrics can be used for optimization of future antennas, i.e., maximizing accessibility in certain pattern regions with the constraint of achieving a target average.

## 5. Conclusions

This paper has introduced a novel methodology for the definition of GNSS constellation antenna patterns with the objective of shortening and simplifying the information transfer from the system to the final SSV user. The proposed procedure has been successfully implemented for the processing of on-ground isolated antenna measurements of Galileo FOC antenna patterns, leading to the definition of a Galileo Reference Antenna Pattern model, recently published in [8]. The solution introduced aims not only at regulating how the system and user share the pattern information, but also setting the basis for a standard procedure that can potentially be adopted by any GNSS system. The approach directly provides a 3D model at user level, which filters out discontinuities and separates what is best considered uncertain due to test-specific realizations and measurement degradation effects. Providing a statistical distribution in the form of confidence bounds, the system informs the user community about the regions or the specific points where performance can deviate from nominal values. The mathematical details of the antenna pattern reconstruction procedure have been presented herein to better understand the GRAP model properties with respect to the objectives of improved resolution, smoothness, and more general representation of the expected constellation EIRP. The approach and its statistical framework could also be considered a fundamental step towards updating GNSS antenna data processing to the state of the art of antenna characterization techniques and statistical learning. The statistical model developed can be considered as a-priori knowledge that can be exploited in the future for new data monitoring, prediction, and integration.

The additional results based on simplified geometrical analysis demonstrate how the GRAP model can be exploited for final Galileo SSV characterization. A new metric has also been introduced, accessibility, which can potentially also be adopted for other GNSS systems to better assess their SSV peculiarities. This index expresses the fraction of the rising GNSS SV orbit arc that the user can potentially exploit to access an SV signal for a target minimum received power. The approach does not pretend to replace standard assessments, which directly derive the GNSS SVs availability and navigation performance alongside the simulated space user trajectory. Accessibility has been proposed as a more compact and faster tool allowing the user to get general information about service quality for a target mission altitude and system or user requirements. In fact, the analysis presented for the Galileo E1 identifies different Galileo SSV operative regions, which reflect different ways to span the GRAP antenna patterns at different altitudes. The transition from one region to another is mainly driven by the capability of the user to access antenna pattern side-lobes for the target mission altitude, hence accessibility can be considered as an effective tool for the preliminary design of the on-board spaceborne GNSS system. It is also not excluded that, even limited by its assumptions, this compact and scalar index can be used as a key performance indicator for the design or adjustment of next-generation GNSS constellation antenna patterns. Small modifications in antenna pattern can correspond to relevant changes in accessibility. As such, this could represent a valid starting point for an optimization-based approach able to leverage SSV criticalities.

Future work will fit within the ongoing international effort for the definition of an Interoperable Global Navigation Satellite Systems Space Service Volume [1]. In the next steps, the GRAP model will be exploited to perform additional Galileo and multi-constellation SSV analysis with the possibility of fostering and better designing next-generation missions in high orbit.

## Data Availability

No new data were created or analyzed in this study. Data sharing is not applicable to this article.

## List of Acronyms

AVE	Average
CW	Continuous Wave
dBW	Decibel Watt
dB/Hz	Decibel per Hertz
EIRP	Equivalent Isotropic Radiated Power
FOC	Galileo Full Operational Capability
GEO	Geostationary Earth Obit
GNSS	Global Navigation Satellite System
GRAP	Galileo Reference Antenna Pattern
HEO	High Earth Orbit
KPI	Key Performance Indicator
MEO	Medium Earth Orbit
SH	Spherical Harmonic
SSV	Space Service Volume
SV	Space Vehicle

## Appendix A

A linear representation directly reflects coefficient and random effect variance, but the dB scale is preferred considering the very small values associated with low-gain regions. Therefore, we present the GRAP in dB scale, considering a log-normal transformation which takes the following expression

(A1) $$\begin{array}{c} {G_{f,m}^{R}}_{dB}=G_{ref,dB}+{K\mu }_{z}\\ \mu_{z}={log}_{e}\left(G_{f,m}^{R}\left(\theta ,\varphi \right)\right)-\frac{\sigma_{z}^{2}}{2} \end{array}$$

where $G_{ref,dB}$ is a normalization offset herein considered when the model is compared with reference data and K = $\frac{20}{{log}_{e}10}$. $\mu_{z}$ is corrected by considering the model standard deviation as

(A2) $$\sigma_{z}=\sqrt{{log}_{e}\left(1+\frac{\sigma_{G_{f,\sigma }^{R}\left(\theta ,\varphi \right)}^{2}}{{\left(G_{f,m}^{R}\left(\theta ,\varphi \right)\right)}^{2}}\right)}$$

where $\sigma_{G_{f,\sigma }^{R}\left(\theta ,\varphi \right)}^{2}$ is the variance extracted from the full variance-covariance matrix $G_{f,\sigma }^{R}\left(\theta ,\varphi \right).$ Asymptotic covariance bounds can be defined by the following expression:

(A3) $${CI}_{m,dB}={G_{f,m}^{R}}_{dB}\pm 2K\sigma_{z}$$

## References

[1] International Committee on GNSS (ICG) (2018). The Interoperable Global Navigation Satellite Systems Space Service Volume.

[2] (2015). GPS Space Service Volume Ensuring Consistent Utility Across GPS Design Builds for Space Users.

[3] Marquis W., Reigh D. (2015). The GPS Block IIR and IIR-M Broadcast L-Band Antenna Panel: Its Pattern and Performance. Navigation.

[4] Enderle W., Gini F., Schönemann E., Mayer V. PROBA-3 Precise Orbit Determination Based on GNSS Observations, 32nd ed. Proceedings of the International Technical Meeting of the Satellite Division of The Institute of Navigation: ION GNSS+ 2019.

[5] Moreau M.C., Davis E.P., Carpenter J.R., Kelbel D., Davis G.W., Axelrad PMoreau M.C., Davis E.P., Carpenter J.R., Kelbel D., Davis G.W. Results from the GPS Flight Experiment on the High Earth Orbit AMSAT AO-40 Spacecraft. Proceedings of the ION GPS 2002 Conference.

[6] Shehaj E., Capuano V., Botteron C., Blunt P., Farine P.-A. (2017). GPS Based Navigation Performance Analysis within and beyond the Space Service Volume for Different Transmitters’ Antenna Patterns. Aerospace.

[7] Ji G.-H., Kwon K.-H., Won J.-H. (2021). GNSS Signal Availability Analysis in SSV for Geostationary Satellites Utilizing multi-GNSS with First Side Lobe Signal over the Korean Region. Remote Sens..

[8] Menzione F., Sgammini M., Paonni M. (2024). Reconstruction of Galileo Constellation Antenna Pattern for Space Service Volume Applications.

[9] Mattes S., Rondineau L., Coq L. (2017). Fast Antenna Far Field Characterization via Sparse Spherical Harmonic Expansion. IEEE Trans. Antennas Propag..

[10] Migliore M.D., Soldovieri F., Pierri R. (2005). Far-field antenna pattern estimation from near-field data using a low-cost amplitude-only measurement setup. IEEE Trans. Instrum. Meas..

[11] Behjoo H., Pirhadi A., Asvadi R. (2019). Efficient Spherical Near-Field Antenna Measurement Using Compressive Sensing Method with Sparsity Estimation. IET Microw. Antennas Propag..

[12] Allende-Alba G., Thoelert S. (2020). Reconstructing antenna gain patterns of Galileo satellites for signal power monitoring. GPS Solut..

[13] Allende-Alba G., Thoelert S. (2020). An analysis of the on-orbit performance of Galileo satellite antennas using reconstructed gain patterns. GPS Solut..

[14] Zheng J., Chen X., Liu X., Zhang M., Liu B., Huang Y. (2022). An Improved Method for Reconstructing Antenna Radiation Pattern in a Loaded Reverberation Chamber. IEEE Trans. Instrum. Meas..

[15] Hastie T., Tibshirani R., Friedman J.H., Friedman J.H. (2009). The Elements of Statistical Learning Data Mining, Inference, and Prediction.

[16] Moore K.M., Bloxham J. (2017). The construction of sparse models of Mars’ crustal magnetic field: Sparse models of Mars’ magnetic field. J. Geophys. Res. Planets.

[17] Bertrand Q., Massias M., Gramfort A., Salmon J. Handling correlated and repeated measurements with the smoothed multivariate square-root Lasso. Proceedings of the Annual Conference on Neural Information Processing Systems 2019, NeurIPS 2019.

[18] Fitzmaurice G., Davidian M., Verbeke G., Molenberghs G. (2008). Longitudinal Data Analysis.

[19] Gustavsson S.M., Johannesson S., Sallsten G., Andersson E.M. (2012). Linear Maximum Likelihood Regression Analysis for Untransformed Log-Normally Distributed Data. Open J. Stat..

[20] Galileo Open Service Signal in Space Interface Control Document, Issue 2.1, November 2023. https://www.gsc-europa.eu/.

[21] Kaplan E., Hegarty C. (2017). Understanding GPS/GNSS: Principles and Applications.

